# Tribute to Professor Anthony J. McMichael

**DOI:** 10.3390/children1030457

**Published:** 2014-11-26

**Authors:** Ashwin Swaminathan, Robyn M. Lucas, David Harley

**Affiliations:** 1 National Centre for Epidemiology and Population Health, Australian National University, Corner of Mills and Eggleston Roads, Canberra, Australian Capital Territory 2601, Australia; E-Mails: robyn.lucas@anu.edu.au (R.M.L.); david.harley@anu.edu.au (D.H.); 2 Departments of General Medicine and Infectious Diseases, Canberra Hospital, Yamba Drive, Garran, Canberra, Australian Capital Territory 2605, Australia; 3 Australian National University Medical School, Australian National University, Canberra, Australian Capital Territory 0200, Australia; 4 Telethon Kids Institute, University of Western Australia, 100 Roberts Road, Subiaco, Perth, Western Australia 6008, Australia

**Keywords:** climate change, epidemiology, environment

## Abstract

Emeritus Professor A. J. “Tony” McMichael (1942–2014) was an internationally renowned and pioneering Australian academic and advocate in epidemiology, who was passionate about understanding the influences of the environment on human health. In an illustrious career spanning more than four decades, he made significant contributions to the scientific community and policy discourse—including ground-breaking research related to the health of children. McMichael was a prolific academic writer with over 300 peer-reviewed papers; 160 book chapters and two sole-authored books. However, his outstanding talent was for integrating complex and seemingly unrelated strands from the environmental and health sciences into a cohesive narrative—and highlighting its relevance to lay persons, scientists and governments alike. He was instrumental in validating this nascent field of research and inspiring many others to follow his lead.

Emeritus Professor A. J. “Tony” McMichael (1942–2014) was an internationally renowned and pioneering Australian academic and advocate in epidemiology, who was passionate about understanding the influences of the environment on human health. In an illustrious career spanning more than four decades, he made significant contributions to the scientific community and policy discourse—including two articles in this journal’s Special Issue on the “Impact of Climate Change on Child Health” [1,2].

McMichael completed his medical degree in 1967, but volunteer trips to assist the poor and ill of India and Papua New Guinea helped him to decide that one need not be a “stethoscope-carrying doctor” to be an effective promoter of health [3]. Elected President of the National Union of (University) Students in the politically and culturally charged period of the late 1960s, he demonstrated an early disposition and skill for influencing important societal debates, experiences that would prove crucial in later policy and advocacy roles.

McMichael’s classical epidemiological training commenced with doctoral studies at Monash University, Victoria, where he researched factors influencing the mental health of undergraduate students. He became fascinated (and alarmed) by the global issues of sustainability and relationships between ecosystems and health. He wrote on these issues in a national newspaper column entitled “Spaceship Earth”:

*“The Spaceship Earth notion [was] that we live within this closed system, this little planet, and the damage that we do to the environment around us will have ways of coming back to bite us, and particularly as the scale of that damage begins to increase we will start to see systemic changes on a larger scale that would have wider ranging consequences for human health both now and into the future.”*

[4]

Whilst McMichael’s epidemiological research was not restricted to a specific area of epidemiology or sub-population, some of his most ground-breaking work was directly relevant to children’s health. On return from post-doctoral research in the United States for example, an opportunity arose to investigate the high rates of pregnancy-related complications in the South Australian town of Port Pirie, home to a large lead smelter. Amongst other notable findings, McMichael’s group found a significant inverse association between post-natal infant lead levels and subsequent neuro-cognitive development at age four and later years [5]. This pioneering research significantly influenced the tightening of environmental exposure standards globally and contributed to the accelerated introduction of unleaded petrol.

McMichael was also instrumental in conducting and broadcasting research investigating the adverse effects of passive exposure to smoke, particularly detrimental to children’s respiratory function. His advocacy extended to giving expert testimony in the Federal Court of Australia, which led to a landmark ruling preventing the tobacco industry from making misleading promotional claims [6].

Unarguably his greatest scientific and policy contribution (and legacy) was his pioneering research and untiring communication of the health effects of global climate change. His authoritative book entitled *Planetary Overload: Global Environmental Change and Human Health (1993)* first alerted the world of the looming dangers of a rapidly changing climate, and led to key roles on influential international bodies, including the Intergovernmental Panel on Climate Change (IPCC) and World Health Organisation.

McMichael was a prolific academic writer with over 300 peer-reviewed papers, 160 book chapters and two sole**-**authored books. However, his outstanding talent was for integrating complex and seemingly unrelated strands from the environmental and health sciences into a cohesive narrative—and highlighting its relevance to lay persons, scientists and governments alike. He was instrumental in validating this nascent field of research and inspiring many others to follow his lead.

For his many and varied contributions, McMichael has been lauded nationally and internationally, including winning the prestigious National Health and Medical Research Council Australia Fellowship (2007), election as a Member of the US National Academy of Sciences (2011) and appointment to the title of Officer of the Order of Australia (2011). His contribution to the IPCC was also commended on the awarding of the 2007 Nobel Prize for Peace to that organisation.

It is fitting that we should leave the last words of this tribute to the man himself. The following is an excerpt from an open letter authored by McMichael (just weeks before his passing) along with prominent national public health figures, to the Australian Prime Minister with an exhortation for climate change to be discussed at the recently concluded G20 Heads of Government Meeting that took place in Australia:

“In the long run, the harm to human health from climate change is more than an avoidable burden of suffering, injury, illness and premature death. It signals that our mismanagement of the world’s climate and environment is weakening the foundations of health and longevity.

*This issue warrants urgent consideration at the G20 meeting. The health of present and future generations is at risk from ongoing human-induced climate change.”*

[7]

Dr Ashwin Swaminathan
Professor Robyn Lucas
Associate Professor David Harley
